# Three-dimensional honeycomb-like porous carbon derived from corncob for the removal of heavy metals from water by capacitive deionization†

**DOI:** 10.1039/c7ra10689k

**Published:** 2018-01-04

**Authors:** X. F. Zhang, B. Wang, J. Yu, X. N. Wu, Y. H. Zang, H. C. Gao, P. C. Su, S. Q. Hao

**Affiliations:** Department of Chemical Engineering, Chengde Petroleum College Xueyuan Road Chengde China zxfzcg168@163.com; College of Material Science and Chemical Engineering, Harbin Engineering University Harbin China yujing006@yeah.net; School of Chemistry, University of Manchester Oxford Road Manchester M13 9PL UK

## Abstract

In this study, porous carbon (3DHPC) with a 3D honeycomb-like structure was synthesized from waste biomass corncob *via* hydrothermal carbonization coupled with KOH activation and investigated as a capacitive deionization (CDI) electrode material. The obtained 3DHPC possesses a hierarchal macroporous and mesoporous structure, and a large accessible specific surface area (952 m^2^ g^−1^). Electrochemical tests showed that the 3DHPC electrode exhibited a specific capacitance of 452 F g^−1^ and good electric conductivity. Moreover, the feasibility of electrosorptive removal of chromium(vi) from an aqueous solution using the 3DHPC electrode was demonstrated. When 1.0 V was applied to a solution containing 30 mg L^−1^ chromium(vi), the 3DHPC electrode exhibited a higher removal efficiency of 91.58% compared with that in the open circuit condition. This enhanced adsorption results from the improved affinity between chromium(vi) and the electrode under electrochemical assistance involving a non-faradic process. Consequently, the 3DHPC electrode with typical double-layer capacitor behavior is demonstrated to be a favorable electrode material for capacitive deionization.

## Abstract 2

A porous carbon electrode with a 3D honeycomb-like structure demonstrates a high removal efficiency for the removal of chromium(vi) from water.

## Graphical abstract

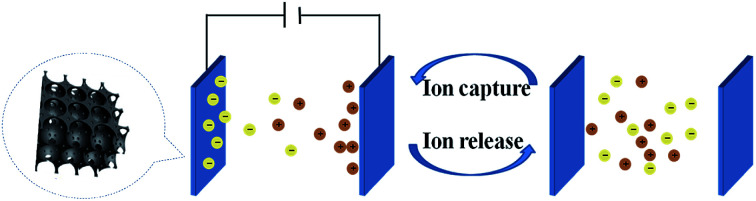

## Introduction

Heavy metals ions, as major components of water pollutants, cause increasing risks to the ecological environment and human health due to their toxicity, pervasiveness, and persistence.^1–3^ In particular, chromium(vi), which is released from plating waste and tanneries, among other industries, if enriched in the human body, can cause irreversible renal injury, muscular cramp, skeletal deformity, and erythrocyte destruction.^4,5^ Thus, the effective recovery of chromium(vi) from waste water has become a crucial issue. Thus far, several methods have been developed to remove chromium(vi) ions from water, including chemical precipitation, membrane filtration, solvent extraction, electrodialysis, and recently, capacitive deionization (CDI).^6–8^ Among these methods, capacitive deionization has emerged as a robust, cost-effective, and energy-efficient competitor to membrane filtration and electrodialysis, with applications ranging from heavy metal removal to water purification.^9–11^

CDI is an electrosorption process that uses a low electrical field to remove ions from solution by adsorbing them onto the surface or into the pores of porous electrodes to form electric double-layer capacitors (EDLCs).^12–14^ At present, the key feature of CDI is to design new electrode materials that exhibit high specific areas, reasonable pore size distributions and good electric conductivity.^15–17^ Besides, chemical stability and cost are significant issues. To date, various types of carbon have been explored as candidates for CDI electrode materials, including activated carbon,^18^ carbon nanotubes,^19,20^ carbon nanofibers,^21,22^ and graphene.^23–26^ Although carbon nanofibers, carbon nanotubes and carbon aerogels have excellent electrosorption performances, their complicated synthetic procedures and high costs limit their practical application, especially in water treatment.^27^ Thus, the preparation of low-cost sorbents with a high sorption efficiency for water treatment is the goal of research in this field.

As a type of environmentally friendly renewable resource, biomass, is an attractive raw material for the synthesis of valuable carbon due to its abundance, low cost and excellent properties.^28,29^ To date, various biomass materials such as pomelo peel,^30^ wood,^31^ eggplant^32^ and watermelon peel^33^ have been applied to prepare carbon materials. For example, Zhao *et al.*^33^ prepared MMC-A using watermelon peel as a carbon source, which was developed as an electrode for deionization capacitors to remove NaCl from saltwater solutions. Porada *et al.*^34^ prepared nanoporous heteroatom-doped carbons using biomass-based carbon precursors such as glucose and glucosamine with 2-thiophenecarboxylic acid (TCA) as a sulfur source. Corncob waste is generally used as a food source for livestock and the excess can be burnt. However, burning it can cause air, soil and water pollution. Therefore, converting it into carbonaceous materials represents a good alternative.

Thus, in this work we demonstrate a simple and economic strategy for the preparation of porous carbon (3DHPC) with a 3D honeycomb-like structure from waste biomass corncob *via* hydrothermal carbonization coupled with KOH activation. Its morphology, pore structure and electrochemical performance are characterized *via* scanning electron microscopy, N_2_ adsorption–desorption and electrochemical measurements, respectively. Besides, this unique 3DHPC is used as an electrode material for CDI to remove chromium(vi). Batch-mode electrosorption experiments are also performed to investigate the potential of 3DHPC as an electrode material for enhancing the adsorption of chromium(vi) during the electrosorption process.

## Experimental

### Preparation of samples

Corncob residues were collected from a farm in Hebei. All chemical reagents were of analytical grade and used without further purification.

The raw corncob waste material was crushed, washed with deionized water several times and dried at 100 °C for 24 h. Then, the dried corncob and citric acid solution (0.1 M) were maintained in a sealed, Teflon-lined autoclave at 200 °C for 6 h. The resulting hydrothermal carbon was collected *via* vacuum filtration, and washed several times with deionized water.

The hydrothermal carbon was added to 4 M KOH solution with stirring for 2 h and the soaked for 12 h. The resultant mixture was put into a corundum boat and heated to 350 °C at 5 °C min^−1^ and kept for 1 h under flowing argon, then heated to 700 °C and kept for 2 h. Finally, 3DHPC was obtained by immersing the carbonized product into 1 M HCl for 6 h with a subsequent washing and drying process. For comparison, the calcination temperature was adjusted to 400 °C and 550 °C. The obtained black carbon materials were named 3DHPC-*x*, where, *x* is the calcination temperature. DHC was prepared in the absence of KOH by directly heating the hydrothermal carbon at 350 °C for 1 h and 700 °C for 2 h under an argon atmosphere (5 °C min^−1^).

### Electrochemical removal of chromium(vi)

Electrochemical experiments were carried out on an electrochemical workstation (CHI660E). A three-electrode system was used, which consisted of platinum foil as the counter electrode, a saturated calomel electrode (SCE) as the reference electrode, and a working electrode. The working electrode was prepared by dispersing 80 wt% as-prepared 3DHPC, 10 wt% acetylene black (AB), and 10 wt% polytetrafluoroethylene (PTFE) in ethanol to yield a homogeneous paste. After ultrasonic dispersion, the paste was uniformly coated on nickel foam (1 × 1 cm^2^) and dried at 60 °C for 12 h. The loading of the electrode materials (3DHPC, AB, and PTFE) was controlled to ∼5 mg. Electrochemical impedance spectroscopy (EIS) measurement on the as-prepared 3DHPC was carried out in the frequency range of 100 kHz to 0.01 Hz with an amplitude of 5 mV in 1 M Na_2_SO_4_. Cyclic voltammetry (CV) tests were performed in a potential window of 0–0.8 V (*vs.* SCE) by varying the scan rate from 10 to 100 mV s^−1^. The specific capacitances were obtained according to the following equation:1
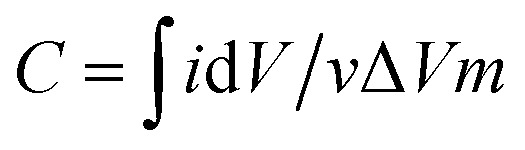
where, *C* is the specific capacitance, *i* is the instant current, d*V* is the potential window, *v* is the scan rate and *m* is the mass of the electrode.

Electroadsorption was carried out in a cuvette with 60 mL of 30 mg L^−1^ chromium(vi) solution. The electrodes consisted of 80 wt% of as-prepared 3DHPC, 10 wt% of acetylene black and 10 wt% of PTFE. The obtained slurries were coated on nickel foam (2 × 2 cm^2^) and dried at 60 °C for 12 h. A range of voltages between 0–2.0 V were then applied to the CDI electrode assembly to investigate the impact of voltage on chromium(vi) removal. In each experiment, the designated voltage was applied to pre-charge the CDI reactor for 10 min before the deionization experiment to ensure that a steady state was reached. The concentration of chromium(vi) ions in the solution was analyzed using the GB method (water quality-determination of chromium(vi): 1,5-diphenylcarbohydrazide spectrophotometric method) and a 722 s spectrophotometer at 540 nm. The removal efficiency and electrosorption capacity were calculated according to eqn (2) and (3):2
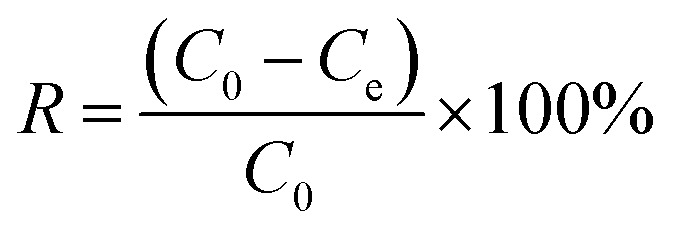
3
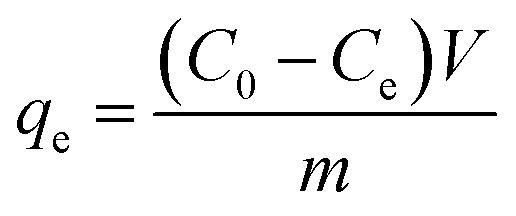
where, *C*_0_ (mg L^−1^) is the initial concentration of chromium(vi), *C*_e_ (mg L^−1^) is the equilibrium concentration of chromium(vi), *V* (L) is the volume of electrolyte and *m* is the weight of active 3DHPC on the electrode (g).

### Characterization

X-ray diffraction (XRD) analysis was performed on a Rigaku D/max-IIIB diffractometer with Cu Kα irradiation (*λ* = 1.54178 Å). The X-ray source was operated at 40 kV and 150 mA. Morphology was characterized *via* scanning electron microscopy (SEM, Hitachi S-4800). Fourier-transform infrared (FT-IR) spectra were recorded with an AVATAR 360 FT-IR spectrophotometer using standard KBr pellets. The BET specific surface area and pore size distribution of the as-prepared samples were measured with a fully automatic surface area analyzer (Quantachrome 2010e) using the nitrogen isothermal adsorption–desorption method.

## Results and discussion

### Characterization of samples

**Fig. 1:**
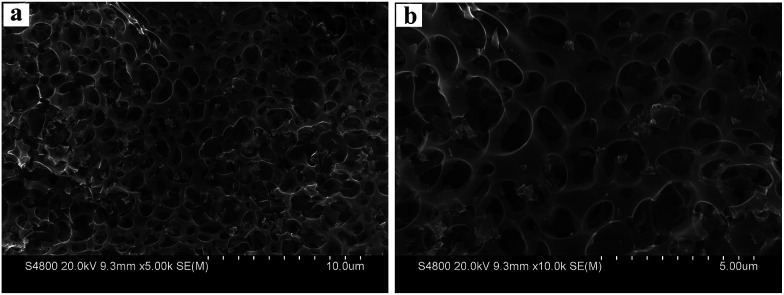
Different magnification SEM images of 3DHPC.

Scanning electron microscopy (SEM) was used to investigate the structure and morphology of the as-prepared material. Fig. 1 shows that the as-prepared 3DHPC has a typical honeycomb-like structure constituted by 3D interconnected carbon walls. The diameter of the macroporous cores is around 600 nm. From the TEM image (Fig. S1, ESI†), it is obvious that 3DHPC shows a macroporous architecture, which is consistent with the SEM image. In contrast, DHC presents a bulk morphology and it is difficult to find any pore structure (Fig. S2, ESI†). During the synthetic process, the KOH particles played an important role in the formation of this unique structure. With annealing, the following reaction occurs between KOH and C.^35^2C + 6KOH → 2K + 3H_2_ + 2K_2_CO_3_

Metallic K and other K compounds could act as a hard template to generate macropores after their removal by diluted hydrochloric acid/deionized water washing, which could act as ion-buffering reservoirs for electrolyte ions, ensuring the rapid transport and diffusion of electrolyte ions and effectively shortening the diffusion paths.^36–38^ Moreover, KOH can etch the dense carbon framework to generate mesopores, which significantly increase the specific surface area and provide more effective electroactive sites, consequently leading to a greatly improved removal efficiency of metal ions.^38^

The activation parameters have great influence on the specific surface area and pore structure. Table 1 exhibits the surface texture properties of all the samples. The specific surface area, pore volume and average pore radius of 3DHPC are much higher than that of DHC. After activation by KOH, the specific surface area and pore volume increased. We attribute the improvement in the specific surface area and pore volume to the effect of KOH. Moreover, with an increase in temperature, the specific surface area and pore volume are enhanced correspondingly. The reason for this is that at higher temperature, the activation process occurs more completely.^33^ In addition, when the temperature is increased, the decomposition rate becomes quicker, which promotes the enlarging of the pore size.^39^ Considering that 3DHPC has a large specific surface area and a large amount of pores, we chose it to study the electrochemical and deionization performance.

**Table 1:** Surface texture properties of all the samples

Characteristic	DHC	3DHPC	3DHPC-550	3DHPC-400
Surface area (m^2^ g^−1^)	513	952	445	357
Pore volume (cm^3^ g^−1^)	0.25	0.51	0.29	0.24
Average pore radius (nm)	1.93	2.96	2.61	2.04

**Fig. 2:**
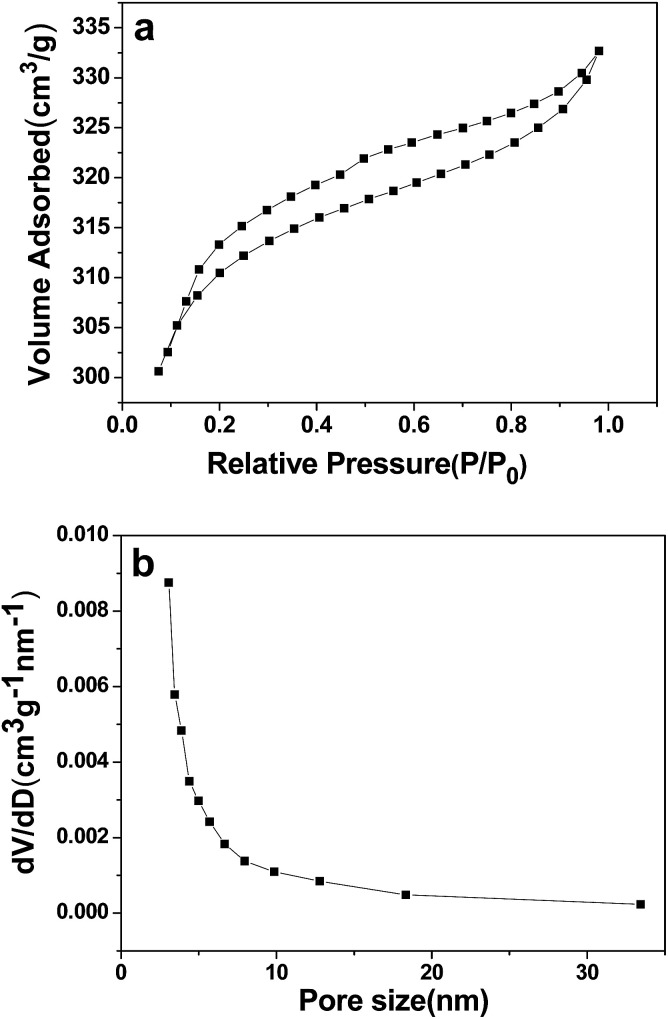
N_2_ adsorption–desorption isotherm (a) and pore size distribution (b) of 3DHPC.

Fig. 2a shows the nitrogen adsorption–desorption isotherm, and Fig. 2b shows the BJH pore-size distribution curve for the as-synthesized 3DHPC. 3DHPC exhibits an isotherm that can described as a mixture of type II and type IV with a type H3 hysteresis loop, according to the IUPAC technical report.^40^ According to the SEM and nitrogen adsorption–desorption curve analysis, it is clear that meso- and macropores coexist in this sample. The electrosorption capacity depends strongly on the pore structure of the carbon electrodes. The macropores can form ion-buffering reservoirs and shorten the ion diffusion distance and thus accelerate the transportation of the ions.^41^ The mesopores can reduce the resistance for the ions in porous carbon electrodes, and the micropores can increase the specific surface area and provide more adsorption sites.^42,43^ This hierarchal macroporous and mesoporous structure with high specific surface area and pore volume could minimize the ion diffusion distance and benefit ion transporting into the inner channels of 3DHPC.

**Fig. 3:**
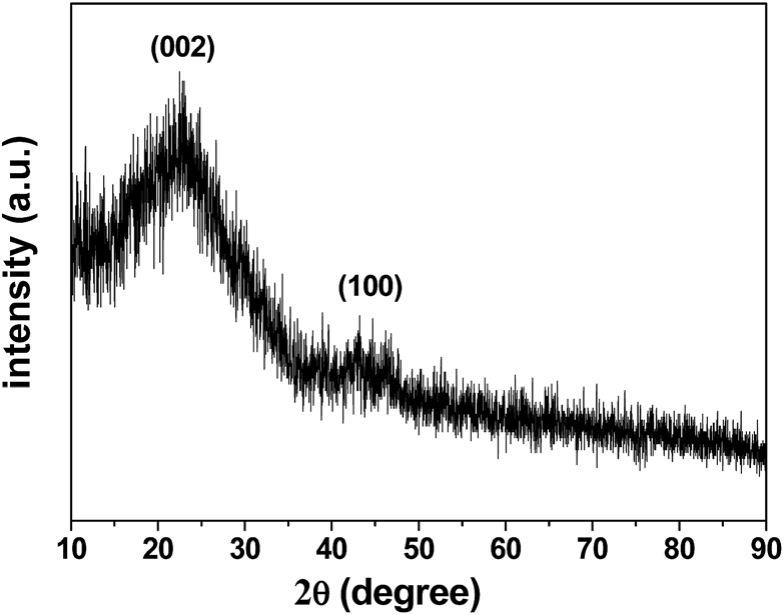
XRD pattern of the as-prepared 3DHPC.

The X-ray diffraction (XRD) pattern of the 3DHPC is shown in Fig. 3. It shows that 3DHPC possesses broad diffraction peaks at 22.7° and 43.0° corresponding to the (002) and (100) reflections, respectively, which indicate an amorphous nature and low graphitization.^44,45^

**Fig. 4:**
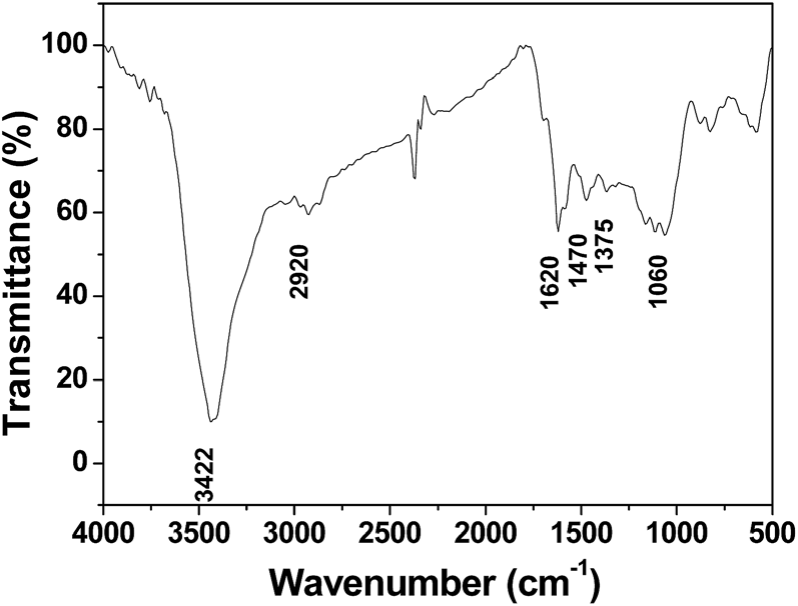
FT-IR spectrum of 3DHPC.

The product was further characterized *via* FTIR spectroscopy. As shown in Fig. 4, the adsorption peak at 3422 cm^−1^ can be assigned to the stretching vibrations of hydroxyl groups. The adsorption peaks at 1620 cm^−1^ and 1060 cm^−1^ are attributed to the C=O and C–O stretching vibrations, respectively. The absorption band observed at 2920 cm^−1^ is assigned to the C–H stretching vibration. The bands at 1470 and 1375 cm^−1^ are mainly due to the C–H bending vibrations. These results demonstrate that oxygen functional groups exist in 3DHPC.

**Fig. 5:**
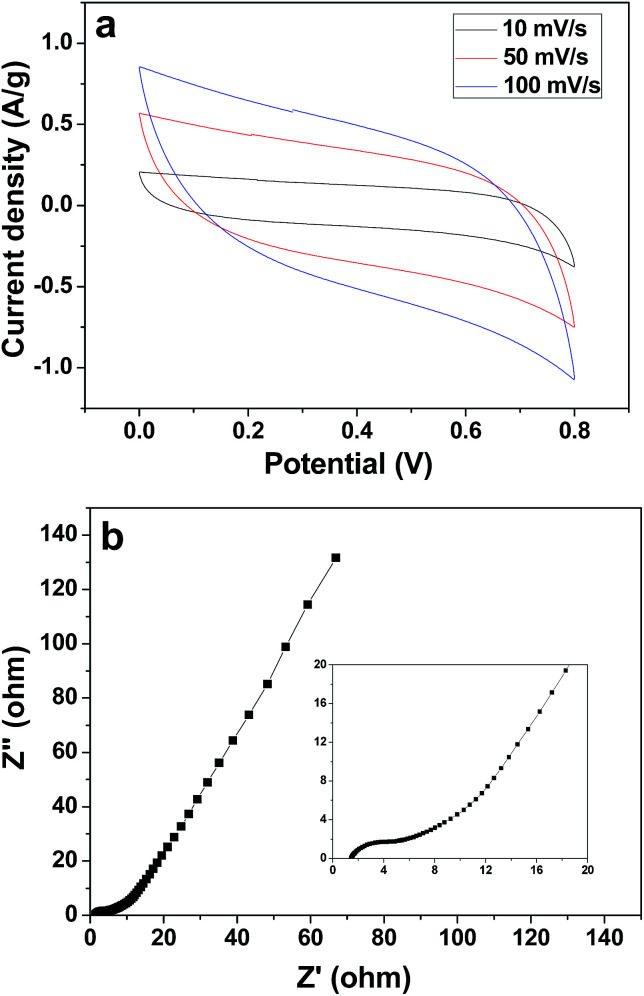
CV curves for 3DHPC at various scan rates (a) and Nyquist plot for the 3DHPC electrode (the inset chart shows the magnified high frequency region) (b).

The electrochemical capacitive property of the corncob-derived carbon material was studied on a three-electrode system, and the electrolyte was 1 M Na_2_SO_4_ aqueous solution (Fig. 5a). At a scan rate of 10 mV s^−1^, the capacitance of the 3DHPC electrode was found to be 452 F g^−1^, which then decreased slightly with an increase in scan rate. This indicates the high charge storage ability and high rate capability of the 3DHPC electrode. Additionally, the near-rectangular CV curve, in which no oxidation/reduction peaks are observed, indicates that the electrode is a typical double-layer capacitor.^46,47^ Moreover, 3DHPC still presents a rectangular CV shape at a high scan rate of 100 mV s^−1^, which implies efficient charge transfer and electrolyte diffusion within the porous carbon.^48^

EIS is an effective tool to examine the resistance characteristic of 3DHPC. Fig. 5b shows the Nyquist plot of the 3DHPC electrode in the frequency range of 0.01–100 kHz, including a quasi-semicircle at the high frequency region and a linear part at the low frequency region. The intercept at the real axis corresponds to the equivalent series resistance, which is mainly responsible for the electrolyte solution resistance, the electrical resistance of the electrode, and contact resistance.^49^ As seen in the inset of Fig. 5b, at high frequency a low intercept with *Z*′ and small semicircle diameter are observed, which indicates a small equivalent series resistance and low charge transfer resistance.^50^ The low equivalent series resistance and charge transfer resistance illustrate that the 3DHPC electrode provides a suitable pore architecture for the formation of an electrical double layer and therefore a high CDI efficiency can be achieved.

### Electrochemical removal of chromium(vi)

**Fig. 6:**
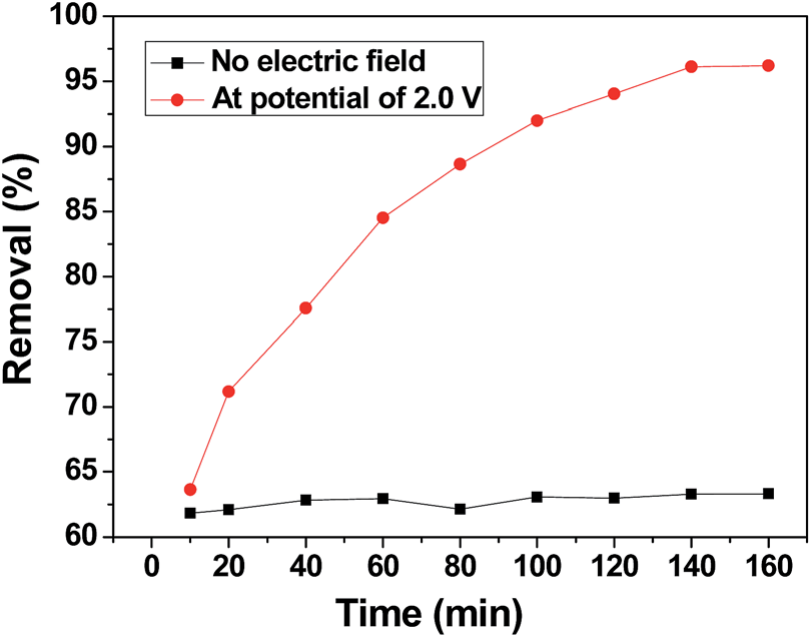
Adsorption and electrosorption removal efficiencies of chromium(vi) on the 3DHPC electrode.

To distinguish the role of adsorption and electrosorption played in chromium(vi) removal, the adsorption of chromium(vi) by the 3DHPC electrode and its electric induced sorption of chromium(vi) under a 2.0 V applied voltage were investigated. As depicted in Fig. 6, without any applied voltage, the removal efficiency of chromium(vi) by the 3DHPC electrode is much lower than that with an applied voltage. The removal of chromium(vi) is mainly accomplished *via* physical or chemical adsorption. With an applied voltage of 2.0 V, the removal efficiency of chromium(vi) by the 3DHPC electrode is dramatically increased to 96.20%. This enhancement in removal efficiency is mainly due to the electrochemical polarization of 3DHPC, which contributes to the capture of chromium(vi) by the opposite charge on the electrode surface.^51^

According to these results, it can be concluded that compared with adsorption, electro-enhanced adsorption of chromium(vi) can be achieved at a low positive potential using the 3DHPC electrode, which results from the improved affinity between chromium(vi) and the electrode. Therefore, this demonstrates the feasibility of the electrosorptive removal of chromium(vi) from aqueous solutions using the 3DHPC electrode.

We investigated the parameters that affect the chromium(vi) adsorption properties of the 3DHPC electrode, including applied voltage and electrolyte concentration were investigated.

### Effect of applied voltage on chromium(vi) removal

**Fig. 7:**
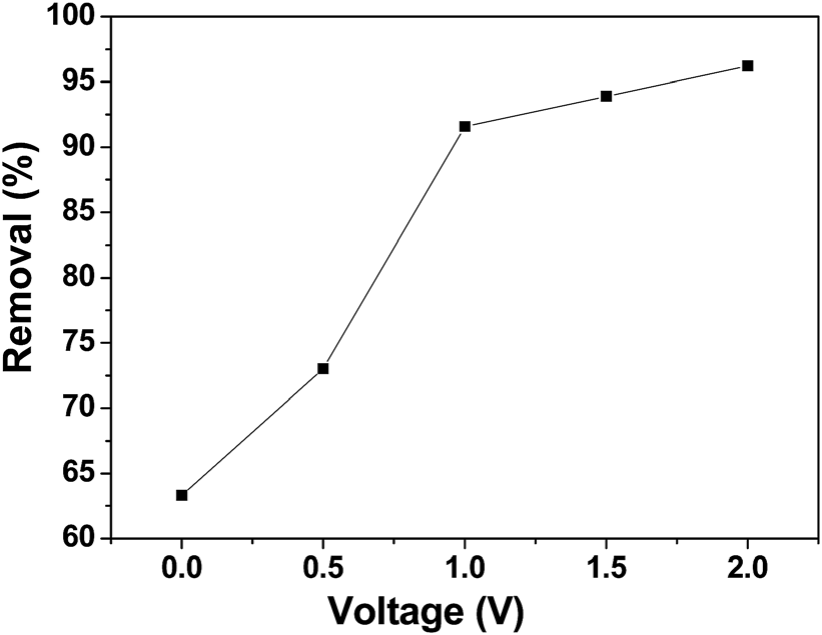
Chromium(vi) removal efficiency of the 3DHPC electrode at different applied voltages.

CDI experiments were conducted to evaluate the effect of voltage on the removal efficiency of chromium(vi) using the 3DHPC electrode. As shown in Fig. 7, the removal efficiencies under 0 V, 0.5 V, 1.0 V, 1.5 V and 2.0 V were 63.31%, 73.01%, 91.58%, 93.90% and 96.20%, respectively, which are positively correlated with the applied electrical forces. Notably, the removal efficiency depends strongly on the voltage, where a higher voltage leads to a higher chromium(vi) removal efficiency. This is most likely because the enhanced electric field elevated the driving force for chromium(vi) absorption as the applied voltage increased. It is also noteworthy that when the potential exceeds 1.23 V, the bonds between the hydrogen and the oxygen atoms break, leading to the electrolysis of water.^52^ Under this condition, more electrical energy is wasted. Hence, the applied voltage of 1.0 V was selected.

### Effect of electrolyte concentration

**Fig. 8:**
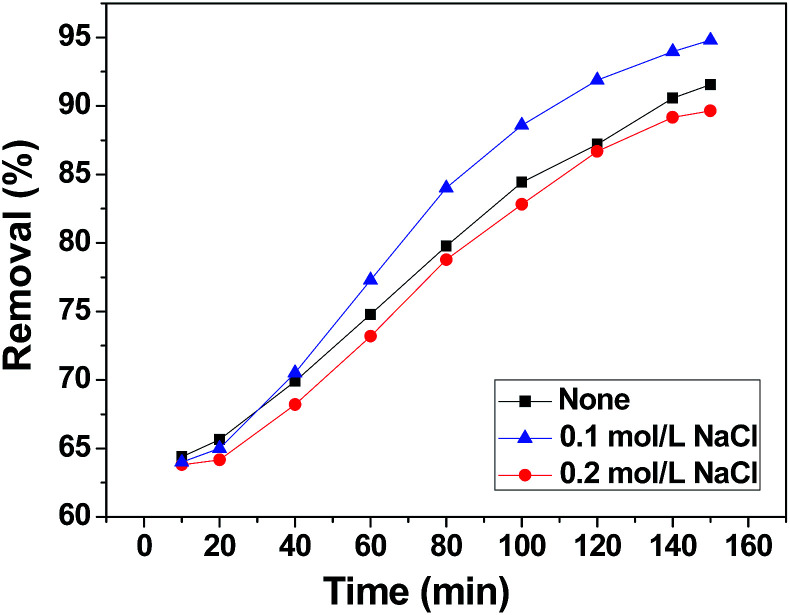
Chromium(vi) removal efficiency of the 3DHPC electrode at different electrolyte concentrations.

The effect of background ions is very important in practical application. In order to adjust the solution electrolyte concentration, NaCl was used as a representative background electrolyte to study the effect of electrolyte concentration on the adsorption of chromium(vi) on 3DHPC. The result is shown in Fig. 8. It obvious that 3DHPC has a lower removal efficiency when the electrolyte is 0 M NaCl than that in 0.1 M NaCl, which may be because increasing the electrolyte concentration increases the conductivity of the solution. When the electrolyte is 0.2 M NaCl, the removal efficiency of chromium decreases, which may be due to the competitive adsorption between Cl^−^ and Cr_2_O_7_^2−^. Cl^−^ ions occupy the adsorption sites on the electrode, resulting in a lower removal efficiency of chromium.

### Kinetic study

**Fig. 9:**
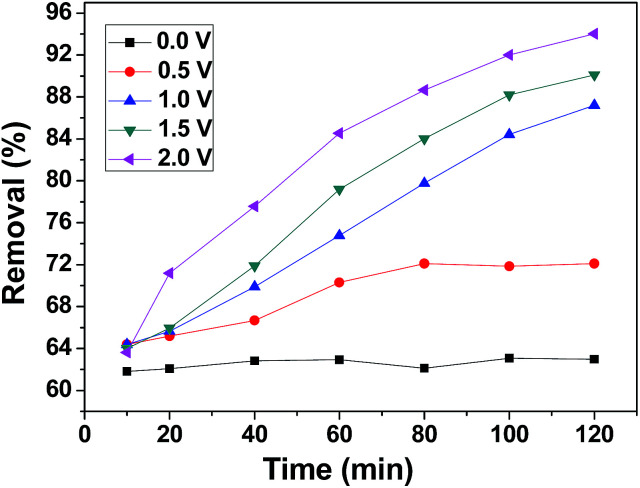
Effect of contact time on chromium(vi) removal.

Fig. 9 presents the kinetics curves of the 3DHPC electrode towards chromium(vi) ions using 60 mL of chromium(vi) (30 mg L^−1^). The rate is rapid during the initial stages of the process. Subsequently, the uptake rate slowly declines with time and tends to equilibrium. The reason for this is that in the initial stages of the process, more target chromium(vi) provides a higher driving force to facilitate ion diffusion from the solution to the active sites. With the occupation of the active sites and the decrease in chromium(vi) concentration, the uptake rate decreases until equilibrium is achieved.^53,54^

Moreover, the electroadsorption process has a longer equilibrium time than the adsorption process. In the process of electroadsorption, this could be related to superficial groups and the ionic mobility of chromium(vi) as a result of the applied voltage. The latter effect gives rise to an increase in the number of contacts between the active sites and chromium(vi) ions. Hence, the equilibrium time for electroadsorption is longer than for adsorption. Meanwhile, the removal efficiency of chromium(vi) that underwent electroadsorption is significantly higher than in the adsorption process.

The experimental data were further simulated using pseudo-first-order and pseudo-second-order models.^55–57^ The pseudo-first-order kinetic equation is given as:

4 ln(*q*_e_ − *q*_*t*_) = ln *q*_e_ − *k*_1_*t*

where, *k*_1_ is the rate constant, and *q*_e_ and *q*_*t*_ (mg g^−1^) refer to the amount of ions adsorbed at equilibrium and at time (*t*), respectively.

The pseudo-second kinetic model developed by Ho and McKay^58^ can be represented by the following equation:5
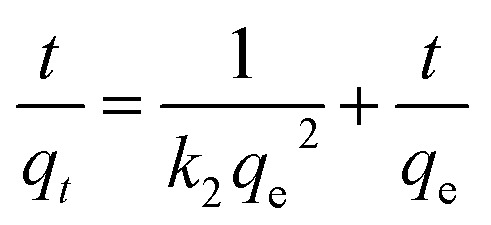
where, *k*_2_ is the rate constant of pseudo-second-order adsorption.

**Fig. 10:**
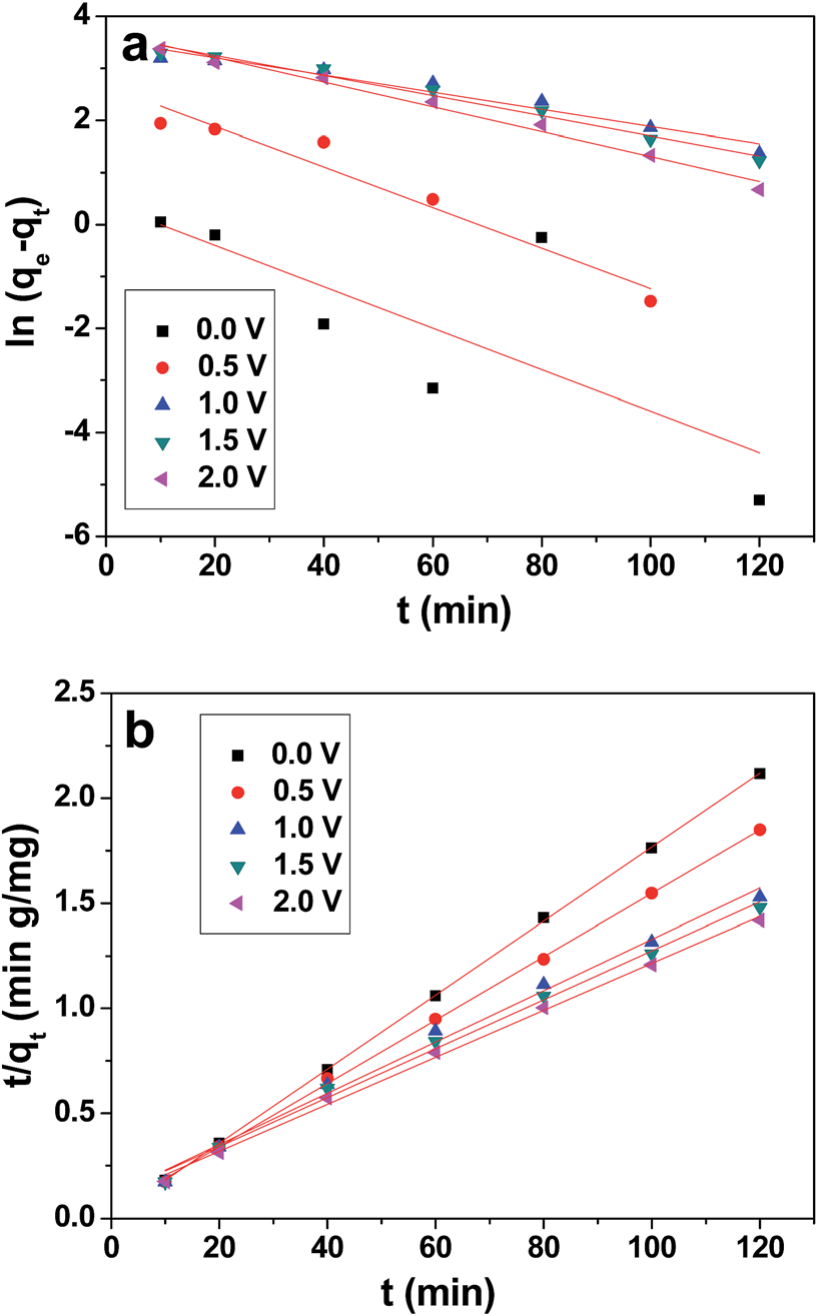
Pseudo-first-order (a) and pseudo-second-order (b) plot for the removal of chromium(vi) by 3DHPC.

The values of *q*_e_, *k*_1_ and *k*_2_ were calculated from the intercepts and slopes values of the plot (Fig. 10a and b), which correspond to eqn (4) and (5), respectively, and are listed in Table 2. It is found that the calculated equilibrium adsorption capacities from the pseudo-second-order model are very close to the experimental data. Moreover, all the regression constants (*R*^2^) for the pseudo-second-order model are better than 0.99, which suggests that the adsorption and the electrosorption process can be well-described by the pseudo-second-order model. The adsorption capacity of chromium(vi) increased when the supplied voltage increased from 0 V to 2.0 V. This is because at a higher supplied voltage the system has a higher driving force, and thereby the adsorption capacity is enhanced. Furthermore, this can be explained by the adsorption mechanism, which involves valence forces through the shared use or exchange of electrons between the chromium(vi) ions and the electrode.^59^ The electrosorption is due to the electrostatic interaction between the ions on the electrode and in solution and valence forces through shared electrons between the chromium(vi) ions and the electrode.^60^

**Table 2:** Pseudo-first and pseudo-second-order constants and values of *R*^2^ for the 3DHPC electrode

Kinetic models and parameters	0 V	0.5 V	1.0 V	1.5 V	2.0 V
*q*_e_ (exp) (mg L^−1^)	56.68	64.89	82.40	84.51	86.58

**Pseudo-first-order**					
*q*_e_ (cal) (mg L^−1^)	1.485	14.39	34.20	37.94	40.04
*k*_1_ (min^−1^)	0.0399	0.0390	0.0165	0.0193	0.0238
*R* ^2^	0.489	0.930	0.947	0.978	0.985

**Pseudo-second-order**					
*q*_e_ (cal) (mg L^−1^)	56.72	66.26	81.83	85.76	89.29
*k*_2_ (g mg^−1^ min^−1^)	0.0627	0.0060	0.0014	0.0013	0.0013
*R* ^2^	0.999	0.999	0.992	0.994	0.997

**Fig. 11:**
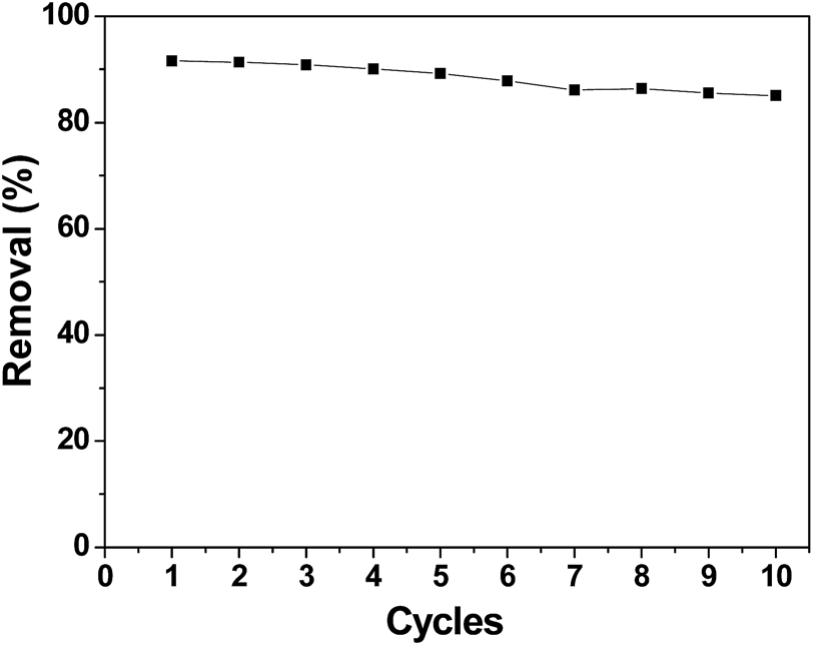
Cycle stability of the 3DHPC electrode.

A good regeneration performance of an electrode material is very important for CDI applications. Fig. 11 shows the removal efficiency of chromium(vi) during the first ten cycles, which was conducted by repeating the adsorption and desorption processes. After ten cycles, the electrosorption capacity did not significantly decrease. The removal efficiency decreased from 91.58% in the first cycle to 85.01% in the tenth cycle. Apparently, the performance of the 3DHPC electrode is not appreciably deteriorated after repeated use. Thus, it can be concluded that the synthesized 3DHPC can be used economically in real processes such as industry wastewater treatment.

Table 3 summarizes previous reports on carbon-based materials as electrodes for chromium adsorption.^60–62^ It is found that 3DHPC as an electrode material for chromium adsorption presents high removal efficiency for chromium(vi). In this regard, the double-layer adsorption theories state that the extent of removal efficiency is governed primarily by the electrostatic attraction force between the ions and the electrode, which is affected by the applied voltage, specific surface area, pore volume and ion solution concentration.^63,64^ Pseudocapacity depends strongly on the chemical characteristics of the solute and the type of bonding between the adsorbate with the adsorbent.^65,66^

**Table 3:** Various carbon materials and their chromium adsorption performances

Electrode material	Surface area (m^2^ g^−1^)	Applied voltage (V)	Initial concentration (mg L^−1^)	Removal efficiency (%)	Ref.
Activated carbon (commercial)	—	1.2	10	97.1	60
	—	1.2	100	42	
Microporous activated carbon prepared from tea	—	1.2	10	88.5	61
SWCNTs@SSNE	380	1.0	6.39	7.8	62
	380	2.5	6.39	99.6	
3DHPC	952	1.0	30	91.58	This work

## Conclusions

Porous carbon with a 3D honeycomb-like structure was successfully synthesized using waste biomass corncob as the raw material *via* hydrothermal carbonization coupled with KOH activation. The resulting 3DHPC possesses a hierarchical porous structure with a high surface area (952 m^2^ g^−1^), and a large pore volume (0.51 cm^3^ g^−1^). This unique 3DHPC was used as an electrode material for CDI to remove chromium(vi) from water. The 3DHPC-based CDI electrode demonstrated a high removal efficiency for the removal of chromium(vi) from water. Thus, this work provides a new method for the efficient removal of chromium(vi) ions from wastewater.

## Conflicts of interest

There are no conflicts to declare

## Supplementary Material

RA-008-C7RA10689K-s001

## References

[cit1] Badr E. A. E., Agrama A. A. E., Badr S. A. E. (2011). Nutr. Food Sci..

[cit2] Cheng S., Grosse W., Karrenbrock F., Thoennessen M. (2002). Ecol. Eng..

[cit3] Järup L. (2003). Br. Med. Bull..

[cit4] Kumar P. A., Ray M., Chakraborty S. (2007). J. Hazard. Mater..

[cit5] Hu J., Chen C., Zhu X., Wang X. (2009). J. Hazard. Mater..

[cit6] Fu F. L., Wang Q. (2011). J. Environ. Manage..

[cit7] Duru I., Ege D., Kamali A. R. (2016). J. Mater. Sci..

[cit8] Hua M., Zhang S. J., Pan B. C., Zhang W. M., Lv L., Zhang Q. X. (2012). J. Hazard. Mater..

[cit9] Oren Y. (2008). Desalination.

[cit10] Forrestal C., Xu P., Ren Z. (2012). Energy Environ. Sci..

[cit11] Liu L. J., Guo X. R., Tallon R., Huang X. K., Chen J. H. (2017). Chem. Commun..

[cit12] Huang Z., Lu L., Cai Z. X., Ren Z. J. (2016). J. Hazard. Mater..

[cit13] Liu P. Y., Wang H., Yan T. T., Zhang J. P., Shi L. Y., Zhang D. S. (2016). J. Mater. Chem. A.

[cit14] Bai Y., Huanga Z. H., Yu X. L., Kang F. (2014). Colloids Surf., A.

[cit15] Porada S., Weinstein L., Dash R., van Der Wal A., Bryjak M., Gogotsi Y., Biesheuvel P. (2012). ACS Appl. Mater. Interfaces.

[cit16] El-Deen A. G., Boom R. M., Kim H. Y., Duan H. W., Chan-Park M. B., Choi J. H. (2016). ACS Appl. Mater. Interfaces.

[cit17] Wen X. R., Zhang D. S., Shi L. Y., Yan T. T., Wang H., Zhang J. P. (2012). J. Mater. Chem..

[cit18] Lu C., Liu C., Rao G. P. (2008). J. Hazard. Mater..

[cit19] Zhang D., Shi L., Fang J., Dai K. (2007). J. Mater. Sci..

[cit20] Wang L., Wang M., Huang Z. H., Cui T. X., Gui X. C., Kang F. Y., Wang K. L., Wu D. H. (2011). J. Mater. Chem..

[cit21] Wang G., Pan C., Wang L., Dong Q., Yu C., Zhao Z., Qiu J. (2012). Electrochim. Acta.

[cit22] El-Deen A. G., Barakat N. A., Khalil K. A., Kim H. Y. (2014). New J. Chem..

[cit23] Li Z., Song B., Wu Z. K., Lin Z. Y., Yao Y. G., Moon K. S., Wong C. P. (2015). Nano Energy.

[cit24] Chen C. C., Yu F., Zhou H. M., Chen J. H., Ma J. (2015). Chemical Journal of Chinese Universities.

[cit25] Duan H. Y., Yan T. T., Chen G. R., Zhang J. P., Shi L. Y., Zhang D. S. (2017). Chem. Commun..

[cit26] Liu P. Y., Yan T. T., Zhang J. P., Shi L. Y., Zhang D. S. (2017). J. Mater. Chem. A.

[cit27] Chen B., Ma Q. L., Tan C. L., Lim T. T., Huang L., Zhang H. (2015). Small.

[cit28] Hou X. X., Deng Q. F., Ren T. Z., Yuan Z. Y. (2013). Environ. Sci. Pollut. Res. Int..

[cit29] Zhu X. D., Liu Y. C., Zhou C., Zhang S. C., Chen J. M. (2014). ACS Sustainable Chem. Eng..

[cit30] Xu D., Tong Y., Yan T. T., Shi L. Y., Zhang D. S. (2017). ACS Sustainable Chem. Eng..

[cit31] Dehkhoda A. M., Ellis N., Gyenge E. (2016). Microporous Mesoporous Mater..

[cit32] Li B., Geng D., Lee X. S., Ge X., Chai J., Wang Z., Zhang J., Liu Z., Hor T. S. A., Zong Y. (2015). Chem. Commun..

[cit33] Zhao S. S., Yan T. T., Wang Z., Zhang J. P., Shi L. Y., Zhang D. S. (2017). RSC Adv..

[cit34] Porada S., Schipper F., Aslan M., Antonietti M., Presser V., Fellinger T. P. (2015). ChemSusChem.

[cit35] Armandi M., Bonelli B., Geobaldo F., Garrone E. (2010). Microporous Mesoporous Mater..

[cit36] Zheng X. Y., Lv W., Tao Y., Shao J. J., Zhang C., Liu D. H., Luo J. Y., Wang D. W., Yang Q. H. (2014). Chem. Mater..

[cit37] Yu P., Zhang Z., Zheng L., Teng F., Hu L., Fang X. (2016). Adv. Energy Mater..

[cit38] Shan D. D., Yang J., Liu W., Yan J., Fan Z. J. (2016). J. Mater. Chem. A.

[cit39] Deng J., Xiong T., Xu F., Li M., Han C., Gong Y., Wang H., Wang Y. (2015). Green Chem..

[cit40] Thommes M., Kaneko K., Neimark A. V., Olivier J. P., Rodriguez-Reinoso F., Rouquerol J., Sing K. S. W. (2015). Pure Appl. Chem..

[cit41] Zhao S. S., Yan T. T., Wang H., Zhang J. P., Shi L. Y., Zhang D. S. (2016). ACS Appl. Mater. Interfaces.

[cit42] Liu P. Y., Yan T. T., Shi L. Y., Park H. S., Chen X. C., Zhao Z. G., Zhang D. S. (2017). J. Mater. Chem. A.

[cit43] Wang H., Yan T. T., Liu P. Y., Chen G. R., Shi L. Y., Zhang J. P., Zhong Q. D., Zhang D. S. (2016). J. Mater. Chem. A.

[cit44] Chen Y. P., Peng L., Zeng Q. R., Yang Y., Lei M., Song H. J., Chai L. Y., Gu J. D. (2015). Clean Technol. Environ. Policy.

[cit45] Fan Y., Yang X., Zhu B., Liu P. F., Lu H. T. (2014). J. Power Sources.

[cit46] Hatzell K. B., Hatzell M. C., Cook K. M., Boota M., Housel G. M., McBride A., Kumbur E. C., Gogotsi Y. (2015). Environ. Sci. Technol..

[cit47] Shi K. Y., Zhitomirsky I. (2015). RSC Adv..

[cit48] Sun L., Tian C., Li M., Meng X., Wang L., Wang R., Yin J., Fu H. (2013). J. Mater. Chem. A.

[cit49] Zhao Y., Hu X. M., Jiang B. H., Li L. (2014). Desalination.

[cit50] Wang H., Shi L., Yan T., Zhang J., Zhong Q., Zhang D. (2014). J. Mater. Chem. A.

[cit51] Han Y. H., Quan X., Zhao H. M., Chen S., Zhao Y. Z. (2007). Front. Environ. Sci. Eng..

[cit52] AlMarzooqia F. A., Al Ghaferia A. A., Saadata I., Hilalb N. (2014). Desalination.

[cit53] Gong J. M., Liu T., Wang X. Q., Hu X. L., Zhang L. Z. (2011). Environ. Sci. Technol..

[cit54] Ai Z. H., Cheng Y., Zhang L. Z., Qiu J. R. (2008). Environ. Sci. Technol..

[cit55] Ho Y. S., McKay G. (2000). Water Res..

[cit56] Yurdakoc M., Scki Y., Yuedakoc S. K. (2005). J. Colloid Interface Sci..

[cit57] Frost R. L., Daniel L., Zhu M. H. Y. (2007). J. Colloid Interface Sci..

[cit58] Ho Y. S., McKay G. A. (1998). Institution of Chemical Engineers.

[cit59] Macías-Garcíaa A., Gómez Corzo M., Alfaro Domínguez M., Alexandre Franco M., Martínez Naharro J. (2017). J. Hazard. Mater..

[cit60] Gaikwad M. S., Balomajumder C. (2017). Sep. Purif. Technol..

[cit61] Gaikwad M. S., Balomajumder C. (2017). Chemosphere.

[cit62] Liu Y. X., X.Yuan D., Yan J. M., Li Q. L., Ouyang T. (2011). J. Hazard. Mater..

[cit63] Schultze J. W., Rolle D. (2003). J. Electroanal. Chem..

[cit64] Farmer J. C., Bahowick S. M., Harrar J. E., Fix D. V., Martinelli R. E., Vu A. K., Carroll K. L. (1997). Energy Fuels.

[cit65] Bizzotto D., Yang Y., Shepherd J. L., Stoodley R., Agak J., Stauffer V., Lathuilliere M., Akhtar A. S., Chung E. (2004). J. Electroanal. Chem..

[cit66] Foo K. Y., Hameed B. H. (2009). J. Hazard. Mater..

