# Supplementary material for: Three-dimensional honeycomb-like porous carbon derived from corncob for the removal of heavy metals from water by capacitive deionization
Source: RSC Adv. 2018 Jan 4;8(3):1159–67. doi: 10.1039/c7ra10689k (PMC9076976; doi:10.1039/c7ra10689k)
Supplement: RA-008-C7RA10689K-s001 [file RA-008-C7RA10689K-s001.pdf]

Electronic Supplementary Material (ESI) for RSC Advances.
This journal is © The Royal Society of Chemistry 2017

Supporting Information

Three-dimensional honeycomb-like porous carbon derived from corn cob for removal of heavy metal from water by capacitive deionization

X. F. Zhang,[a b*] B. Wang,[c] J. Yu,[b*] X. N. Wu,[a] Y. H. Zang,[a] H. C. Gao,[a] P. C. Su[a] and S. Q. Hao[a]

[a] *Department of Chemical Engineering, Chengde Petroleum College, Xueyuan Road, Chengde, China. E-mail: zxfzcg168@163.com*

[b] *College of Material Science and Chemical Engineering, Harbin Engineering University, Harbin, China. E-mail: yujing006@yeah.net*

[c] *School of Chemistry, University of Manchester, Oxford Road, Manchester M13 9PL, United Kingdom*

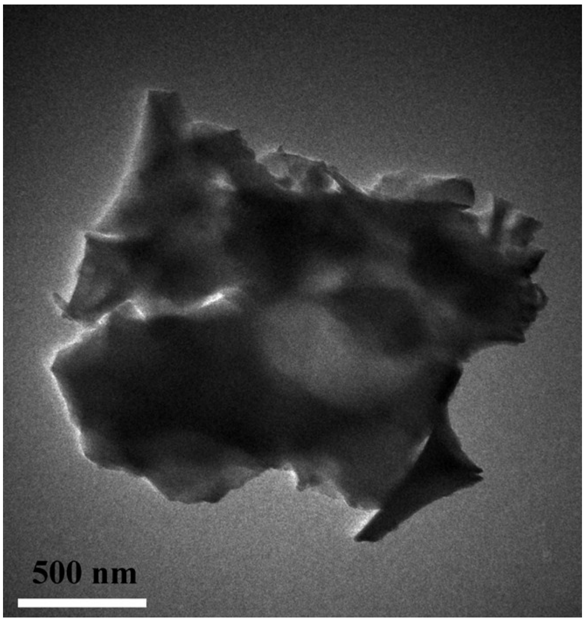

Figure S1. TEM image of 3DHPC.

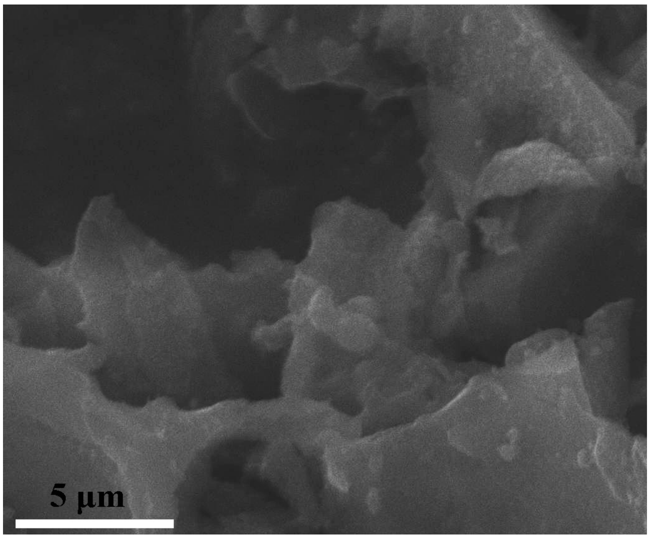

Figure S2. SEM image of DHC.
